# Integrated PBPK-EO modeling of osimertinib to predict plasma concentrations and intracranial EGFR engagement in patients with brain metastases

**DOI:** 10.1038/s41598-024-63743-z

**Published:** 2024-06-03

**Authors:** Feng Liang, Yimei Zhang, Qian Xue, Xiaoling Zhang

**Affiliations:** Bethune International Peace Hospital, Shijiazhuang, China

**Keywords:** Osimertinib, PBPK-EO model, Appropriate dosing regimens, DDIs, Clinical pharmacology, Pharmacokinetics

## Abstract

The purpose of this study was to develop and validate a physiologically based pharmacokinetic (PBPK) model combined with an EGFR occupancy (EO) model for osimertinib (OSI) to predict plasma trough concentration (C_trough_) and the intracranial time-course of EGFR (T790M and L858R mutants) engagement in patient populations. The PBPK model was also used to investigate the key factors affecting OSI pharmacokinetics (PK) and intracranial EGFR engagement, analyze resistance to the target mutation C797S, and determine optimal dosing regimens when used alone and in drug-drug interactions (DDIs). A population PBPK-EO model of OSI was developed using physicochemical, biochemical, binding kinetic, and physiological properties, and then validated using nine clinical PK studies, observed EO study, and two clinical DDI studies. The PBPK-EO model demonstrated good consistency with observed data, with most prediction-to-observation ratios falling within the range of 0.7 to 1.3 for plasma AUC, C_max_, C_trough_ and intracranial free concentration. The simulated time-course of C797S occupancy by the PBPK model was much lower than T790M and L858R occupancy, providing an explanation for OSI on-target resistance to the C797S mutation. The PBPK model identified ABCB1 CL_int,u_, albumin level, and EGFR expression as key factors affecting plasma C_trough_ and intracranial EO for OSI. Additionally, PBPK-EO simulations indicated that the optimal dosing regimen for OSI in patients with brain metastases is either 80 mg once daily (OD) or 160 mg OD, or 40 mg or 80 mg twice daily (BID). When used concomitantly with CYP enzyme perpetrators, the PBPK-EO model suggested appropriate dosing regimens of 80 mg OD with fluvoxamine (FLUV) itraconazole (ITR) or fluvoxamine (FLUC) for co-administration and an increase to 160 mg OD with rifampicin (RIF) or efavirenz (EFA). In conclusion, the PBPK-EO model has been shown to be capable of simulating the pharmacokinetic concentration–time profiles and the time-course of EGFR engagement for OSI, as well as determining the optimum dosing in various clinical situations.

## Introduction

Non-small cell lung cancer (NSCLC) is the most common type of lung cancer, accounting for more than 80% of all cases^1^. Within the NSCLC patient population, the Epidermal Growth Factor Receptor (EGFR) has emerged as a promising therapeutic target^2^. EGFR mutations play a crucial role as oncogenic driver alterations in NSCLC, occurring in about 10–15% of cases among Caucasians and at a higher frequency of up to 50% among East Asians^3^. One specific mutation, known as the T790M mutation in exon 20 of EGFR, was first discovered and described in 2004^3^. Moreover, it is worth mentioning that approximately 30% of NSCLC patients also experience brain metastases^4^.

Osimertinib (OSI) is a registered, selective, and irreversible third-generation EGFR inhibitor specifically prescribed for the management of NSCLC patients possessing the EGFR T790M and L858R mutations^5^. OSI undergoes metabolism mainly by the enzyme CYP3A, and there are minor contributions from CYP1A2 and CYP2C9^6^. Additionally, in vitro studies have demonstrated that OSI acts as a substrate for the efflux transporters ABCB1 (P-glycoprotein) and BCRP (also known as ABCG2, breast cancer resistance protein)^7^. The findings of the study revealed that the brain penetration of OSI in knockout mice lacking Abcb1 and Abcg2 transporters was considerably higher compared to that observed in wild-type mice^7^. Furthermore, OSI has exhibited a relatively high ability to penetrate the cerebrospinal fluid (CSF), with concentrations reaching approximately 2.5% of those present in plasma^8^. This notable penetration into brain tissue (BRT) holds the potential to achieve therapeutic concentrations, offering a promising avenue for treating brain metastases. To date, multiple clinical studies have demonstrated the efficacy of OSI in the treatment of patients experiencing intracranial progression and exhibiting the T790M and L858R mutations^9–11^.

Currently, there have been approximately 29 identified types of EGFR mutations^12^. Among these mutations, the C797S mutation accounts for over 20% of reported cases in clinical settings, thereby imparting a heightened resistance to OSI^13^. Notably, the brain emerges as the primary site of disease progression following treatment for NSCLC. In order for an EGFR inhibitor to exhibit efficacy, it must successfully traverse the blood–brain barrier (BBB), reaching the intended target cells at a substantial free concentration. Nevertheless, multiple factors have proven to exert a significant impact on the plasma concentration of OSI, including the activity of ABCB1^14^ and the level of albumin. Several clinical studies have underscored the noteworthy influence of ABCB1 activity and plasma albumin level on the clinical effectiveness of OSI^15,16^.

Several studies have indicated a strong association between the level of kinase engagement and the clinical response rate^17,18^. For instance, a clinical study demonstrated that zanubrutinib achieved close to 100% engagement of BTK at steady-state, which was crucial for achieving a better clinical response^17^. Another study showed that acalabrutinib achieved over 90% occupancy of BTK, resulting in a response rate of over 80%^18^. Therefore, it is plausible to assume that higher intracranial engagement of OSI at steady-state could be correlated with improved clinical efficacy. However, the extent to which larger EGFR engagement translates into clinical efficacy has not been definitively determined. According to the study^19^, it has been proposed that achieving 80% engagement of OSI in BRT may serve as an effective threshold. For kinase inhibitors, the plasma trough concentration (C_trough_) at steady state is often associated with clinical efficacy. However, multiple clinical studies have indicated that there is no clear relationship between exposure and efficacy for OSI within the dose range^16,20^. On the other hand, plasma C_trough_ levels of OSI exceeding 385.0 ng/mL (equivalent to 711 nmol/L) have been associated with a higher incidence of adverse events greater than or equal to Grade 3, leading to 30% of patients discontinuing the clinical study due to serious adverse events^21^. Therefore, it can be suggested that achieving > 80% engagement of OSI in BRT and maintaining a plasma C_trough_ level of < 711 nmol/L can be considered as the threshold values for achieving clinical pharmacology (PD) efficacy and PK safety in NSCLC patients with brain metastases.

The PBPK approach coupled with an EO model (PBPK-EO) was used to predict target occupancy and plasma C_trough_ of OSI in NSCLC patients. The specific objectives of the study were:

i. Predict the plasma C_trough_ of OSI and the level of intracranial EGFR engagement (wild-type, T790M, L858R, and C797S) in a population of NSCLC patients using the PBPK-EO model, as well as explain on-target resistance mechanism to C797S.
ii. Analyze the impact of ABCB1 activity, albumin level, and EGFR expression on the plasma C_trough_ and intracranial EGFR engagement.
iii. Determine the optimal dosing regimen for OSI when administered alone or in combination with perpetrators of five CYP metabolizing enzymes.

## Methods

### Development of PBPK-EO model

In order to predict the plasma C_trough_ and the occupancy of T790M and L858R mutations in BRT, a whole-body PBPK model integrated with an EO model was developed. The PBPK-EO model was constructed using PK-Sim software (Version 11.2, Bayer Technology Services, Leverkusen, Germany). Table 1 summarized the drug-specific parameters, the binding kinetics of OSI to EGFR, as well as the disease-related physiological parameters^22–34^. Supplementary Figure S1 illustrates the compartmental structure of the of PBPK model. For predicting OSI absorption, the weibull absorption model was utilized, characterized by the weibull time and shape parameters^35^. The distribution of OSI to various organs/tissues and cellular permeability calculations were determined using the Rodgers and Rowland method^36^, as well as the standard PK-Sim. To predict the free concentration of OSI in BRT, the BRT-to-plasma concentration ratio (K_BRT,p_) was assigned at 2.89, based on the mean experimental data reported in the literature^24^. Additionally, the organ-to-plasma partition coefficient (K_p_ scale) was optimized to 1.5 to better describe the OSI distribution. One key aspect to highlight is the optimization of four parameters: GET, Weibull time, Weibull shape, and distribution calculation, K_p_ scale. GET and the Weibull parameters are directly associated with absorption, and optimizing them aims to enhance the alignment between predicted and clinically observed peak times of PK profiles. On the other hand, distribution calculation and K_p_ scale are closely related to distribution, and optimizing them is essential for accurately characterizing tissue distribution to predict plasma concentration reliably, especially at the endpoint of the observation period. These optimizations were conducted by comparing the model’s predictions with observed PK data through the parameter identification module in PK-Sim.

**Table 1:** Model input parameters for the PBPK-EO model of OSI.

Parameters		Values	Source and comments	Descriptions
Physicochemical				
MW (g·mol^−1^)		499.6	Chemspider	Molecular weight
pKa (Base)		9.5, 4.4	Ref.^22^	Base dissociation constant
Log P		5.45		Lipophilicity
Solubility (mg·mL^−1^)		3.1 (Water)	Ref.^23^	Solubility in water
f_up_		0.011 (binding to albumin)	Mean value from in vitro data, Refs.^22,24^	Fraction of free drug in plasma
Absorption				
GET (min)		120	Optimized from 190 min in Ref.^25^ to better fit peak time	Gastric emptying time
P_eff_ (× 10^–4^ cm s^−1^)		0.187	Ref.22	Human effective permeability
Weibull time (min)		15.0	Optimized based on quick dissolution and high solubility at pH 1.0–6.8	Dissolution time of 50% OSI
Weibull shape		0.92		Shape parameter of weibull function
Distribution				
Distribution calculation		Rodgers and Rowland	Optimized for better description of OSI tissue distribution	Calculation method from cell to plasma coefficients
		PK-Sim Standard		Permeability calculation method across cell
Rbp		1.0	Ref.^22^	Blood-to-plasma concentration ratio
K_BRT,p_		2.89	Mean value form the Ref.^24^	BRT-to-plasma partition coefficient
K_p_ scale		1.5	Optimized based on better tissue distribution description	Organ-to-plasma partition coefficient
Elimination				
CYP1A2 CL_int,u_ (μL/min/pmol)		0.52	Ref.^22^	Intrinsic clearance for CYP metabolizing enzymes
CYP2A6 CL_int,u_ (μL/min/pmol)		0.37		
CYP2C9 CL_int,u_ (μL/min/pmol)		0.48		
CYP2E1 CL_int,u_ (μL/min/pmol)		0.11		
CYP3A4 CL_int,u_ (μL/min/pmol)		0.73		
CYP3A5 CL_int,u_ (μL/min/pmol)		0.21		
ABCB1 CL_int,u_ (μL/min/million cells^−1^)		73.4	Estimated based on in vitro transport of OSI in MDR1-MDCK and BCRP-MDCK cell monolayers (Ref.^26^)	Intrinsic transport velocity for ABCB1and BCRP, respectively
BCRP CL_int,u_ (μL/min/million cells^−1^)		15.9		
CL_R_ (L/h)		GFR × f_up_	Default calculation in PK-Sim	Renal clearance
Concentration (μM)	ABCB1	1.3	Abundance values were from Ref.^27^, then calculated based on Concentration = abundance × mg protein/g tissue × brain weight	Concentration for ABCB1, ABCG2, and BCRP transporters
	BCRP	2.4		
Interactions				
K_i_ CYP3A4/5 (μM)		2.55	Ref.^22^	Inhibition constant
K_i_ ABCB1		3.8	Ref.^28^	
K_i_ BCRP		2.0	Ref.^22^	
EC_50_ CYP3A4 (μM)		0.12	Ref.^22^	Inducer concentration required to achieve 50% inductive effect
E_max_ CYP3A4		10.8		Maximum inductive effect
EGFR occupancy				
k_on_ EGFR (μM^−1^ s^−1^)	Wild type	0.028	Calculated using k_inact_/K_i_. k_on_ values for wild-type, L858R + T79M,a nd L858R mutations were taken from Ref.^29^. k_inact_ and K_i_ values were taken from Refs.^30,31^	Association rate constant to EGFR
	L858R + T790M	1.40		
	L858R	0.57		
	C797S	0.0026		
k_off_ EGFR (h^−1^)		0.001	Assigned based on covalent binding to EGFR	Dissociation rate constant from EGFR
EGFR T_0_ (μM)		0.299	Ref.^32^	Start concentration of EGFR
k_deg_ EGFR (h^−1^)		0.025	Ref.^33^, k_deg_ = ln (27.5 h), fitted half-life of 27.5 h in cell experiments	Degradation rate constant for EGFR
Physiological				
Hematocrit		0.33	Mean value of 0.33 in patients from the Ref.^34^	Hematocrit
Albumin (g/dL)		0.31	Value in patients from the Ref.^34^	Plasma albumin level

The hepatic and intestinal metabolic clearance of OSI in the PBPK-EO model was described by six cytochrome P450 (CYP) metabolizing enzymes. These enzymes are responsible for the metabolism of OSI in the liver and intestine, affecting its overall clearance from the body. Additionally, the transport of OSI across the BBB from the brain blood to brain cells was taken into account in the model. This transport process involves both passive permeability and active efflux mediated by the ABCB1/BCRP transporters. The passive permeability coefficient for OSI across the BBB was determined to be 1.36 and 0.83 × 10^−6^ cm/s^26^ for ABCB1 and BCRP, respectively.

The active efflux clearance of OSI mediated by ABCB1/BCRP was also considered in the model. The CL_int,u_ values (intrinsic transport velocities) for ABCB1 and BCRP were estimated using previously reported equations^37^:

1 $${CL}_{int,u}=\frac{2\times {P}_{app,A-B}\times \left(NFR-1\right)\times SA}{\gamma \times cells},$$

where P_app,A-B_ (× 10^–6^ cm/s) is apparent permeability from the apical (A) to the basolateral (B) direction for ABCB1 and BCRP transporters in the MDCKII cell monolayers at pH 7.4. The P_app,A-B_ were determined to be 1.36 and 0.83 for ABCB1 and BCRP, respectively based on experimental measurements^26^. NFR is net efflux ratio. In this case, the NFR values for ABCB1 and BCRP were found to be 13.4 and 5.4, respectively^26^. SA is the filter surface area in 12-cell transwell. Cell represents cell amount. λ (unionization efficiency) is estimated by:

2 $${\text{Log}}_{10}\frac{unionized}{ionized}=pH-PKa.$$

The accuracy of the calculated CL_int,u_ values for ABCB1 and BCRP has been verified. The use of ratios of AUC and C_max_ when considering the presence or absence of CL_int,u_, and comparing these with observed ratios between wild-type and ABCB1 and BCRP knockout mice. The results, as provided in Supplementary Table S1, serve as evidence supporting the accuracy of the calculated CL_int,u_ values for both ABCB1 and BCRP.

The EGFR occupancy is calculated by^18^:

3 $$\frac{dEO}{dt}={k}_{on}\times {C}_{OSI,BRT}\times {EGFR}_{free}-{k}_{off}\times EO,$$

4 $$\frac{d{EGFR}_{free}}{dt}=\left({EGFR}_{0}-{EGFR}_{free}\right)\times {k}_{deg}-{k}_{on}\times {C}_{OSI,BRT}\times {EGFR}_{free}+{k}_{off}\times EO,$$

5 $$\text{EGFR occupancy (\%)}=\frac{\left({EGFR}_{0}-{EGFR}_{free}\right)}{{EGFR}_{0}}\times 100,$$

where EO represents concentration of OSI-EGFR complex formed. EGFR_free_ is the concentration of free EGFR mutations (T790M and L858R). EGFR_0_ is initial EGFR expression. C_OSI,BRT_, k_on_ and k_off_ are the free OSI concentration in BRT, association and dissociation rate constant. k_deg_ represents rate constant of EGFR mutations degradation. The covalent and irreversible binding of OSI to EGFR is accounted for by calculating the association rate constant (k_on_) using the ratio of the inactivation rate constant (k_inact_) to the dissociation constant (K_i_). The value of k_off_, which represents the dissociation rate constant, theoretically approaches zero as the binding between OSI and EGFR is considered irreversible. In the model, a small finite value at 0.001 h^−1^ is assigned to k_off_. Drawing from the findings of previous papers^35^, the brain model in PK-Sim is segmented into four sub-compartments: plasma, blood cells, interstitial, and intracellular space. In this study, the interstitial sub-compartment’s concentration is assumed to reflect the free concentration in BRT. Consequently, the C_OSI, BRT_ is simulated utilizing the free concentration in the interstitial sub-compartment. In the PBPK-EO model, the plasma albumin levels play a role in determining the concentration of OSI in patients. The plasma albumin levels were set at 0.45 g/dL in the healthy population and 0.31 g/dL in the diseased population, based on information obtained from published papers^34^. To incorporate the effect of plasma albumin levels on OSI concentration in patients, the model utilizes the plasma protein scale factor (PPSF) in PK-Sim software. The PPSF is estimated using a specific equation^38^:

6 $${\text{PPSF}} = {1}/\left( {{\text{f}}_{{{\text{up}}}} + \left( {{1} - {\text{f}}_{{{\text{up}}}} } \right) \times {\text{albumin}}_{{\text{f}}} } \right),$$

where f_up_ is fraction of plasma free OSI. The albumin_f_ is the fractional value of plasma albumin in healthy subjects than that in patients.

### Validation PBPK-EO model

In the validation of the PBPK-EO model, four published papers^39–42^ were used to assess the accuracy of predicted plasma PK parameters such as area under the curve (AUC), maximum concentration (C_max_), and C_trough_ of OSI. These papers likely provided experimental data on OSI PK in different patient populations and under various conditions that were used to compare with the model predictions. Additionally, five clinical studies^21,43–46^ were utilized to validate the performance of the model in predicting the free concentration of OSI in the BRT. This validation was done by comparing the observed concentrations of OSI in CSF obtained from these studies with the simulated free OSI concentrations in the BRT by PBPK-EO model.

To further validate the model’s predictions, the time-course of dual mutations of T790M/L858R in NCI cells, as observed in a published paper^19^, was used. This data was utilized to assess the accuracy of the model in predicting the time-profiles of T790M/L858R occupancy mediated by OSI.

In the simulations, the virtual population’s demographic characteristics, including race, dosage, subject numbers, age, proportion of females, body mass index (BMI), and albumin levels, were obtained from the respective clinical studies, as mentioned in Table 2. When data was absent, the mean values available in PK-Sim and in the published papers were used as a surrogate for the missing information.

**Table 2:** Dosing regimens and demographic characteristics in the simulations of PBPK-EO model development and validation.

Clinical study	Race and population	Dosage (mg)	Number of subjects	Age range (year)	Proportion of female (%)	BMI (kg/m^2^)	Albumin level	Purpose
Planchard et al.^39^	Japanese, NSCLC	20, 40, 80 16, 240	28	Median 62.5	75	Mean 26.6	–	Develop model with plasma PK
Zhao et al.^40^	Chinese, NSCLC	40	15	33–73	47	16–31	–	Validate plasma PK
		80	16	35–76	69	18–32		
Harvey et al.^41^	White, NSCLC	80	49	44–83	71	Mean 23.0	–	
Grande et al.^42^	White, NSCLC	80	10	56–73	60		2.8–3.5	
Goldstein et al.^43^	Brain metastatic NSCLC	80	11	31–74	46	–	–	Confirm the model’s appropriateness for predicting intracranial PK
Yamaguchi et al.^44^	Japanese, brain metastatic NSCLC	80	40	41–84	70	–	–	Validate intracranial PK
Leeuw et al.^45^	Brain metastatic NSCLC	80	4	61–70	75	–		
Fukuhara et al.^21^	Japanese, metastatic and non-metastatic NSCLC	80	41	43–81	80	–	–	
Ekman et al.^46^	Brain metastatic NSCLC	80	4	50–80	50	–	–	

–: no data reported.

### Sensitivity analysis of modelling parameters

The sensitivity analysis was performed to identify the modeling parameters that could potentially have a significant impact on the predicted C_trough_ of OSI in plasma, C_trough_ in BRT, and the EO_trough_ (trough level of occupied EGFR) for T790M and L858R mutations. The selected modeling parameters for analysis include: f_up_, albumin level, CYP CL_int,u_, CL_int,u_ for ABCB1 and BCRP, k_on_, k_off_, EGFR T_0_, and EGFR k_deg_. For each of these parameters, an alteration of ± 20% was made in the sensitivity analysis. The sensitivity coefficient (SC) was then calculated using the following equation:

7 $${\text{SC}} = \Delta {\text{Y}}/{\text{Y}} \div \Delta {\text{P}}/{\text{P,}}$$

where ∆Y is the alteration of predicted C_trough_ in plasma, C_trough_ in BRT, and EO_trough_; Y is the initial value; ∆P is the alteration of model parameters; P is initial value of parameters. If the absolute value of the SC is above 1.0, it suggests that variations in these parameters by ± 20% would have a notable impact on the model predictions for C_trough_ in plasma, C_trough_ in BRT, and EO_trough_ for T790M and L858R mutations.

### Effect of the several factors on plasma C_trough_ and EO_trough_

Three specific factors were investigated for their influence on plasma C_trough_ and intracranial EO_trough_. Based on insights from studies^47^, a significant four-fold change in efflux ratios was detected among different variants. Consequently, in this study, the CL_int,u_ of OSI mediated by the ABCB1 efflux transporter was set across a spectrum of 2.0–32 μL/min/million cells^−1^. This variation in ABCB1 activity allows for an assessment of its impact on the plasma C_trough_ and intracranial EO_trough_. Drawing on findings from studies^48,49^, it was observed that albumin levels among cancer patients varied significantly, ranging from 0.28 to 5.8 g/dL. Consequently, the range of albumin levels was established between 1.0 and 6.0 g/dL in this simulation. By considering different albumin levels, the model can evaluate how changes in plasma albumin concentration influence the C_trough_ and intracranial EO_trough_ of OSI. The initial EGFR concentration (EGFR T_0_) was varied in the range of 0.06–1.5 μM. This parameter represents the baseline EGFR concentration and by altering it, the model can assess its effect on the predicted plasma C_trough_ and intracranial EO_trough_.

For these simulations, the dosing regimen of OSI was designated as 80 mg once daily (OD) for 14 consecutive days. This allows for the evaluation of C_trough_ and EO_trough_. The virtual population’s demographic characteristics were set to match those of the clinical study conducted by Planchard, as mentioned in Table 2. This ensures that the virtual population characteristics align with the actual patient population used in the clinical study, providing a relevant context for the model evaluation.

### Simulations for optimum dosing regimen when administration alone

Based on the research findings^19^, it has been established that to achieve a sufficient clinical response, the EO_trough_ should be maintained at a minimum of 80% for both T790M and L858R mutations. Additionally, to ensure clinical safety, it is recommended that the plasma C_trough_ of OSI remains below 711 nmol/L^21^. To ensure that EO_trough_ is above 80% and C_trough_ remains below 711 nmol/L in patients, multiple dosage regimens of OSI ranging from 20 to 240 mg OD or twice daily (BID) were incorporated into the PBPK-EO model. In these simulations, the virtual population’s demographic characteristics were set according to the clinical study conducted by Planchard, as outlined in Table 2. To enhance the accuracy of the simulations, a total of 100 virtual patients were included. By running the simulations, the PBPK-EO model can provide predictions of plasma C_trough_ and EO_trough_ for different dosage regimens of OSI. Based on the simulation results, an optimal dosing regimen of OSI for clinical treatment can be proposed. This regimen aims to achieve EO_trough_ levels above 80% and C_trough_ levels below 711 nmol/L, ensuring both efficacy and safety in the treatment of patients with T790M and L858R mutations.

### Simulations of optimum dosing regimen in DDIs

The integration of the developed PBPK-EO model of OSI with PBPK models of itraconazole (ITR), fluconazole (FLUC), fluvoxamine (FLUV), rifampicin (RIF), and efavirenz (EFA) enables the simulation of changes in plasma C_trough_ and EO for T790M/L858R mutations when OSI is co-administered with CYP3A4, CYP1A2, and CYP2C9 perpetrators. The PBPK modeling parameters for the five perpetrators were obtained from the referenced papers^50^, while the inhibition and induction parameters were sourced from the specified papers^50–52^ and are detailed in Supplementary Table S2. In the DDI simulations, the dosing regimens were: (i) OSI was designed as 80 mg OD; (ii) for perpetrators: ITR at 200 mg BID, FLUC at 150 mg OD, FLUV at 50 mg OD, and RIF and EFA at 600 mg OD. Simulations were carried out after the co-administration of the five CYP metabolizing enzymes for 14 consecutive days. To ensure that the simulations are reflective of real-world scenarios, the virtual population’s demographic characteristics were set to match those of the clinical study conducted by Planchard, as indicated in Table 2. To balance computational efficiency with meaningful results, the number of virtual patients in the population was set to 10 to avoid excessively time-consuming calculations. Following the DDI simulations, the PBPK-EO model was utilized to explore the optimal dosing regimen of OSI when co-administered with the CYP enzyme perpetrators. This exploration aimed to identify the most effective and safe dosing regimen of OSI in the presence of these specific DDIs.

## Results

### Validation of PBPK-EO model

**Figure 1:**
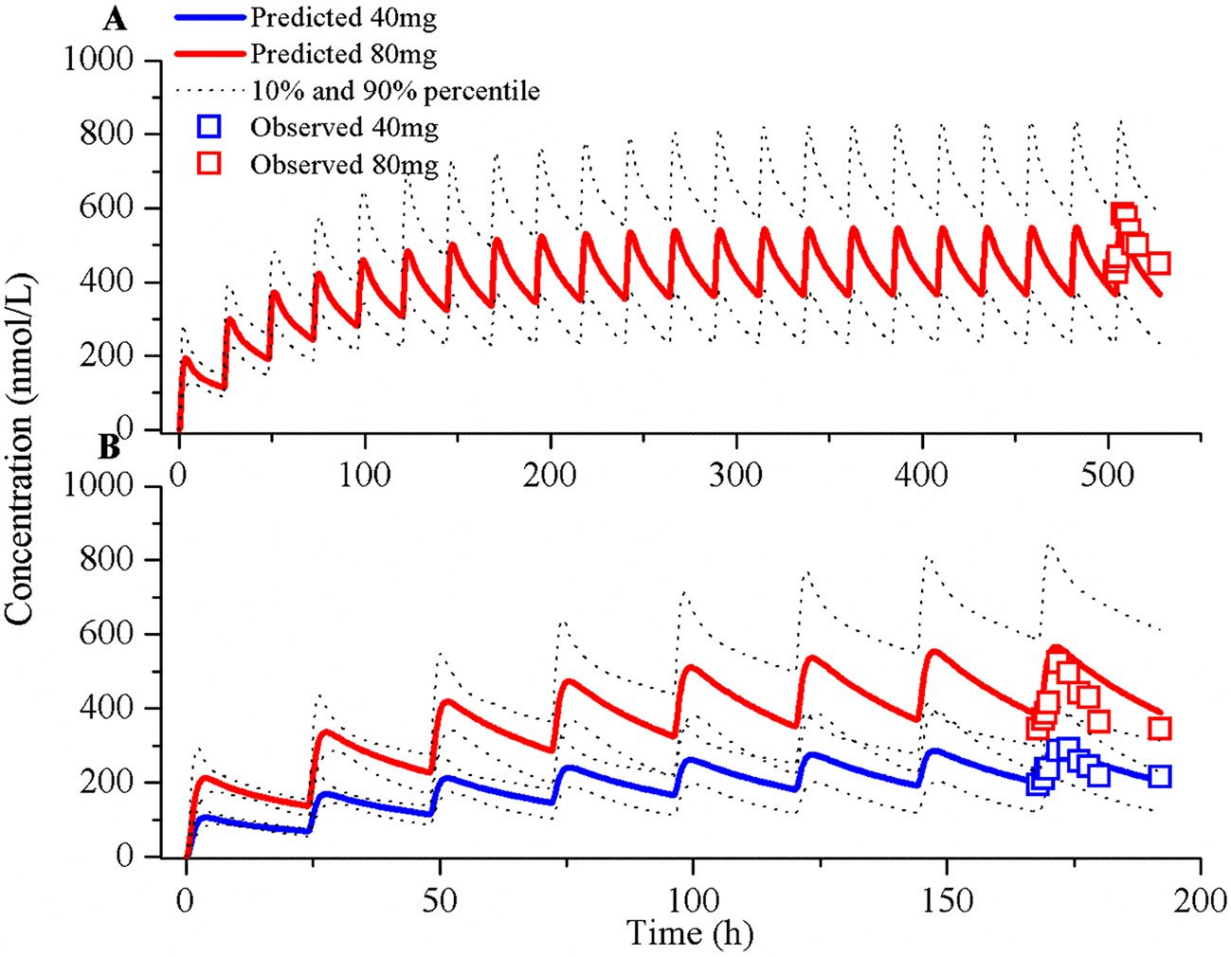
Simulations of the pharmacokinetics of OSI after administration of repeated doses. The simulated and observed plasma concentration–time profiles based on the clinical study by Planchard et al. (**A**) and Zhao et al. (**B**).

Figure 1 displays the predicted and observed plasma concentration–time profiles following oral administration of repeated doses of 40 and 80 mg of OSI in patients. The simulations demonstrate that the population PBPK model effectively replicates the clinically determined PK profiles. In Table 3, comparison of observed PK parameters with the simulated values for OSI is presented. Notably, all the ratios of plasma AUC, C_max_, and C_trough_ fall within the range of 0.5–2.0, with the majority falling within 0.7–1.30. This indicates strong agreement between the simulated PK parameters and the observed values, affirming the accuracy of the model in predicting OSI plasma PK parameters. Moreover, the strong agreement between predicted and observed free concentration values in BRT, with the exception of one data point, further supports the accuracy and robustness of the PBPK model in predicting intracranial PK parameters of OSI.

**Table 3:** Summary of clinical studies used to verify the PBPK-EO model of OSI between predicted and observed PK parameters.

Clinical study	PK	Dosing regimens (mg)	AUC (nmol·h/L, range/CV%^a^)		C_max_ (nmol/L, range/CV%)		C_trough_ (nmol/L, range/CV%)		Prediction/observation ratio		
			Prediction	Observation	Prediction	Observation	Prediction	Observation	AUC	C_max_	C_trough_
Planchard et al.	Plasma	20	2591 (1771–3951)	1964 (871–4990)	134.5 (90.7–205.8)	106.4 (45.4–280.0)	87.2 (539–139.7)	51.2 (21.2–179.0)	1.32	1.26	1.70
		40	5153 (3527–7863)	5640 (2040–14,100)	261.3 (177.0–398.8)	306.2 (127–807)	168.0 (104.0–268.2)	179.3 (58–420)	0.91	0.85	0.94
		80	12,382 (7969–17,551)	11,930 (3650–38,900)	586.8 (378.4–839.8)	623.8 (167–2100)	406.7 (231.6–581.2)	386.4 (104–1440)	1.04	0.94	1.05
		160	26,272 (18,184–39,925)	23,910 (5950–97,000)	1180.7 (808.8–1774.5)	1255 (282–4760)	805.5 (511.9–1264.6)	784.4 (151–3560)	1.10	0.94	1.03
		240	38,188 (24,519–54,062)	28,310 (1150–51,200)	1650.3 (1079.6–2335.1)	1491 (723–2620)	1118.6 (633.8–1590.3)	929.1 (294–1840)	1.35	1.11	1.20
Zhao et al.		40	7105 (34.9%)	5698 (53%)	309.1 (28%)	303.4 (48%)	217.4 (34%)	183.0 (60%)	1.25	1.02	1.19
		80	12,306 (42.6%)	9570 (36%)	598.5 (29%)	550.4 (32%)	377.0 (44%)	318 (43%)	1.29	1.09	1.19
Harvey et al.		80	12,923 (35%)	11,530 (37%)	572.0 (30%)	620.1 (34%)	375.5 (46%)	291.8 (45%)	1.12	0.92	1.29
Grande et al.		80	13,447 (52%)	15,780 (38%)	535.7 (35%)	291.8 (45%)	350.0 (38%)	–	0.85	1.84	–
Goldstein et al.	Intracranial	80	296 (216–389)	–	12.7(9.5–16.6)	–	12.0 (8.3–15.8)	14.4	–	–	0.83
Yamaguchi et al.		80	420 (301–679)	–	18.0 (13.3–28.9)	–	10.3 (6.0–17.6)	4.1 (2.45–8.38)	–	–	2.51
Leeuw et al.		80	235 (170–320)	–	16.8 (10.8–24.6)	–	12.6 (6.5–17.5)	17.5	–	–	0.72
Fukuhara et al.		80	321 (219–421)	–	20.2 (12.3–29.9)	–	15.9(8.0–26.0)	18.3	–	–	0.87
Ekman et al.		80	380 (2078–508)	–	16.2 (11.1–21.5)	–	15.3 (11.0–20.8)	19.5^b^	–	–	0.78

^a^CV%, percentage coefficient of variation.

^b^Calculated based on a ratio of 3.9 between brain and plasma.

–: not reported data.

**Figure 2:**
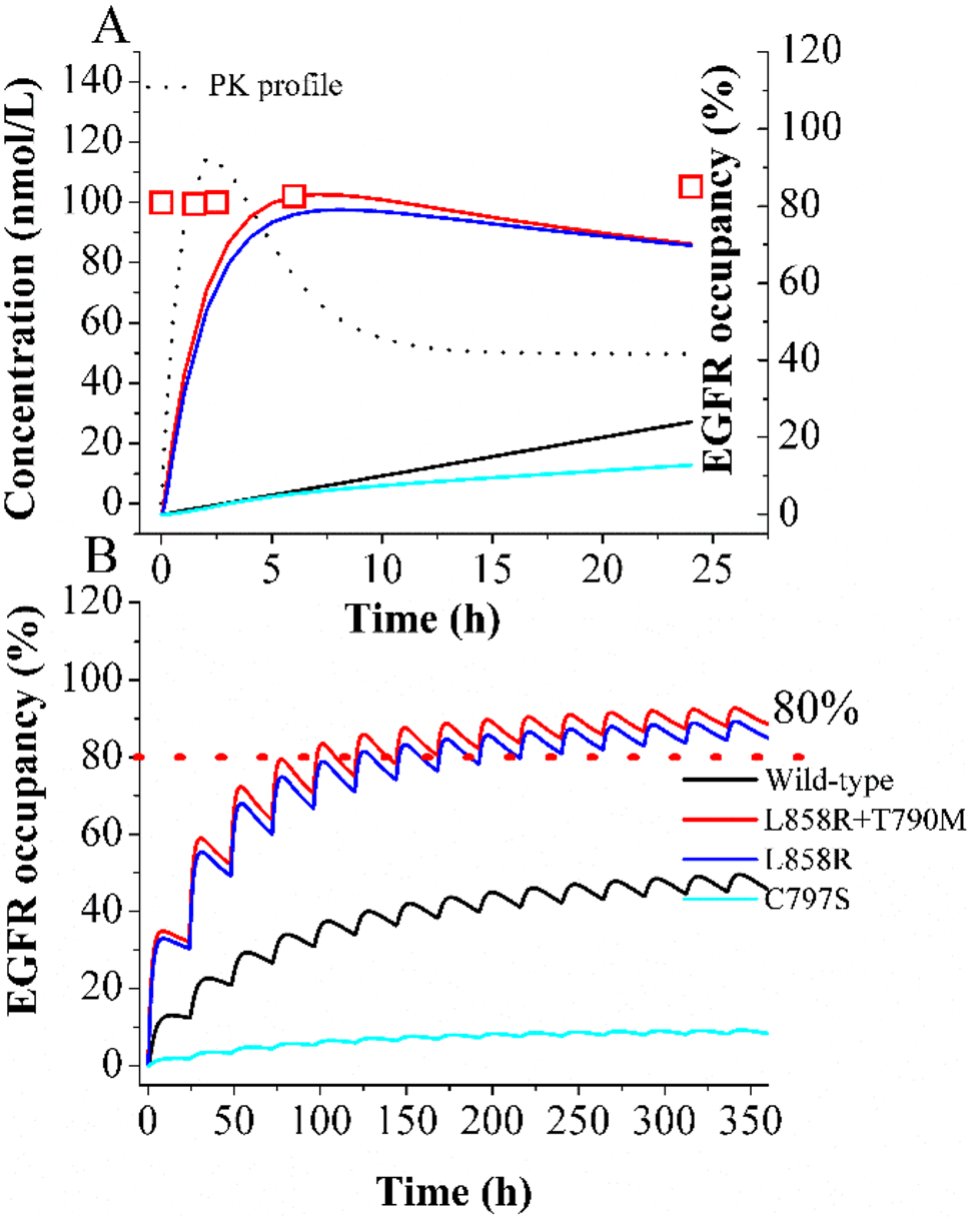
Time-course of intracranial T790M/L858R mutation occupancy by OSI. The time-course of predicted and observed T790M/L858R occupancy in BRT at 100 nmol/L of OSI (**A**). The EO time-profiles by OSI for four type of EGFR after oral administration for consecutive 14 days (**B**).

The simulations of the time course of T790M/L858R dual mutation occupancy in patients by OSI have been depicted in Fig. 2A. The simulation is consistent with the observed time-course of T790M/L858R inhibition in NCI cells at 100 nmol/L. Figure 2B illustrates the time-course of wild-type, T790M/L858R, L858R, and C797 mutations in BRT by OSI. The simulation indicates that the TO_trough_ in brain for T790M/L858R and L858R mutations exceeds 80% at steady-state. This finding suggests that OSI demonstrates high efficacy for patients with brain metastases, as it is able to maintain a high level of intracranial inhibition for these mutations over time. Conversely, the TO_trough_ in BRT for the C797S mutation by OSI is approximately 10%. This value is significantly lower than the effective PD threshold, aligning with the clinically observed resistance of OSI to C797S mutation.

Overall, the simulation results provide valuable insights into the efficacy of OSI in targeting T790M and L858R mutations in brain metastases, as well as resistance to C797S mutation.

### Sensitivity analysis of modelling parameters

The sensitivity analysis presented in Supplementary Table S3 indicates that Albumin level and f_up_ were identified as the sensitive parameters for C_trough_ in plasma and BRT among all the selected parameters. As f_up_ was determined from the in vitro experiments, and Albumin level and f_up_ are highly correlated (see Eq. 6). Hence, subsequent examination of the impact of this modeling parameter on plasma C_trough_ and EO_trough_ in BRT was not carried out. While ABCB1 CL_int,u_ and EGFR T_0_ did not exhibit a significant impact EO_trough_ in the sensitivity analysis, further research was still made to evaluate the effect of these modeling parameters on EO_trough_ in BRT. This decision was influenced by the significant impact of ABCB1 activity on OSI exposure observed in clinical studies^14,15^, as well as the association EGFR expression with worse progression in patients^53^.

### Effect of the several factors on plasma C_trough_ and intracranial EO_trough_

**Figure 3:**
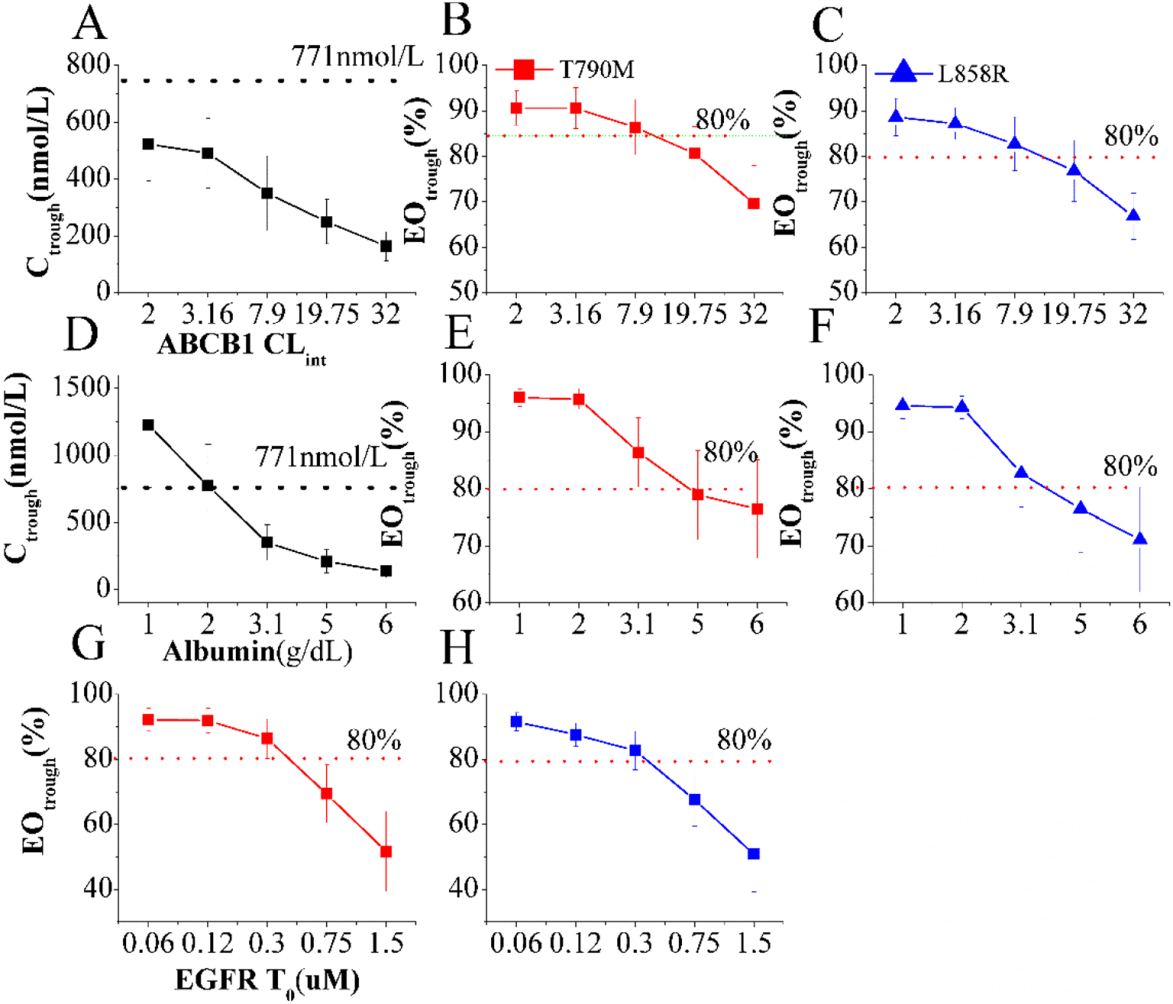
Effect of several factors on plasma C_trough_ and intracranial T790M/L858R occupancy. Plasma C_trough_ and TO_trough_ are affected by ABCB1 C_lint,u_ (**A–C**), albumin level (**D–F**), and EGFR T_0_ (**G,H**).

In Fig. 3A–C, the impact of ABCB1 CL_int,u_ on both plasma C_trough_ and intracranial T79M/L1858R occupancy of OSI is illustrated. The simulations demonstrate that while ABCB1 CL_int,u_ has a notable effect on T79M/L1858R occupancy, it does not exceed the established PK safety threshold for plasma C_trough_. Specifically, an increase of ABCB1 CL_int,u_ in patients by more than 2.0-fold of the original value leads to a reduction of intracranial T79M/L1858R occupancy to below 80%. In Fig. 3D–F, the influence of albumin levels on both plasma C_trough_ and intracranial T79M/L1858R occupancy of OSI is illustrated. The simulations highlight the significant impact of albumin levels on both parameters. A reduction in plasma albumin levels by approximately 0.65-fold compared to original value in plasma C_trough_ exceeding the PK safety threshold. Conversely, an increase in plasma albumin levels by about 1.6-fold compared to original value leads to a reduction of intracranial T79M/L1858R occupancy to below 80%.

Additionally, Fig. 3G,H reveal a substantial impact of EGFR T_0_ on intracranial T79M/L1858R occupancy. The simulations show that the T79M/L1858R occupancy falls outside the range of efficacy PD thresholds when EGFR T_0_ reaches levels of 0.75 and 1.5 μM. These findings underline the significant influence of ABCB1 CL_int,u_, albumin levels, and EGFR T_0_ on the plasma PK and intracranial PD of OSI, providing valuable insights for personalized treatment approaches and patient stratification.

### Simulations for optimum dosing regimen when administration alone

**Figure 4:**
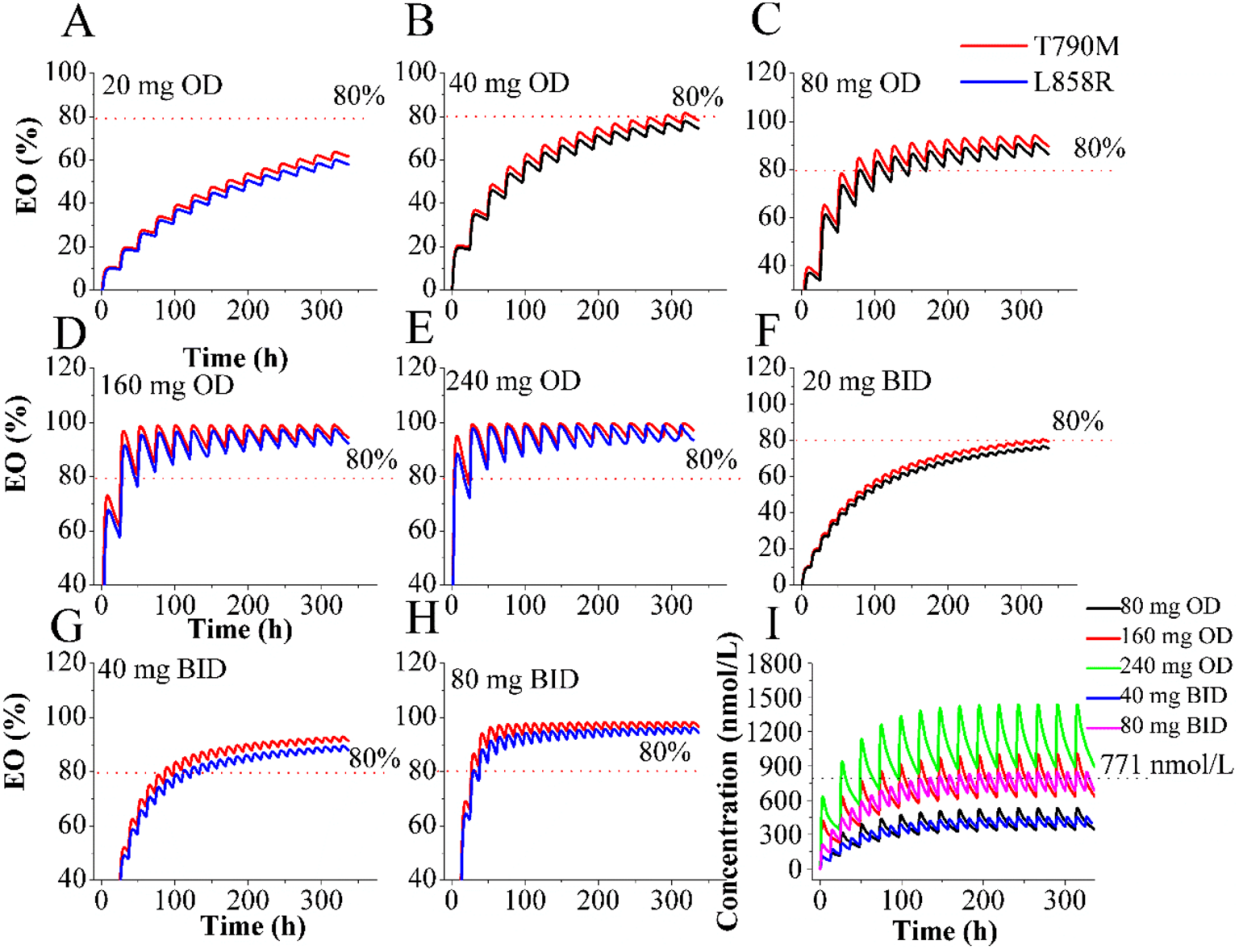
Simulations of time-course of intracranial T790M/L858R and L858R mutations occupancy by OSI. Time-profiles of EO for T790M/L858R and L858R mutations were simulated at different dosing regimens (**A–H**); Plasma concentration–time profiles of OSI were simulated at multiple dosing regimens (**I**).

The Fig. 4A–H provide a visual representation of the time-course of intracranial T790M/L858R and L858R mutations occupancy by OSI at steady-state following oral administration of multiple dosing regimens. The simulations reveal that the T790M/L858R occupancy values remain above 80% for five dosing regimens, indicating sustained target engagement within the brain. However, it is observed that the plasma C_trough_ of OSI exceeds the established PK safety threshold at a dose of 240 mg OD (Fig. 4I). Based on the predictions of the PBPK-EO considerations, it is suggested that dosing regimens of 80 mg and 160 mg OD, as well as 40 mg and 80 mg BID, represent suitable options for the therapy of patients with brain metastases. Furthermore, taking into account administration compliance in the clinical setting, the PBPK-EO model supports that the dosing regimen of 80 mg or 160 mg OD is optimal for achieving clinical efficacy and safety. Notably, these findings align with the dosing regimens examined in multiple clinical trials^11,43^.

Additionally, the model indicates that OSI at 160 mg OD can effectively engage T790M/L858R at higher levels and for a prolonged duration, exceeding the 80% EO threshold, compared to at 80 mg OD. These observations are consistent with data from clinical studies, where dose escalation to 160 mg OD demonstrated modest improvement for patients with brain metastases compared to the 80 mg OD regimen^11^.

### Simulations of optimum dosing regimen in DDIs

**Figure 5:**
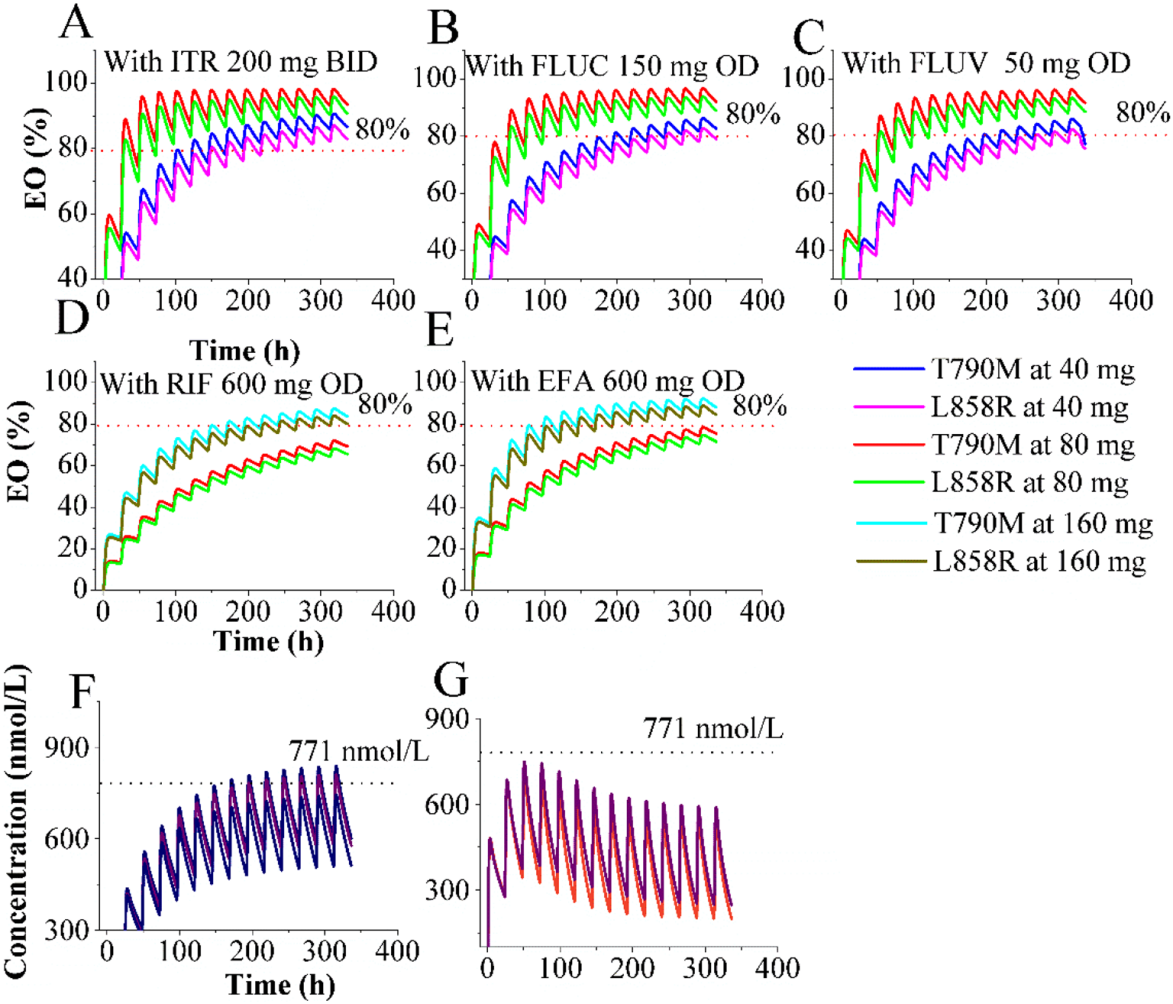
Simulations of time-course of intracranial T790M/L858R and L858R mutations occupancy by OSI in the presence of DDIs. Time-profiles of EO for T790M/L858R and L858R mutations were simulated with five different CYP perpetrators (**A–E**); Plasma concentration–time profiles of OSI were simulated at multiple dosing regimens (**F,G**).

Table 4 summarizes the predicted and observed ratio of plasma AUC and C_max_, demonstrating that the predicted PK parameters from the PBPK-EO model align well with clinically observed data in the DDI simulations^54^. The Fig. 5A–E present the time-course of intracranial T790M/L858R and L858R mutations occupancy by OSI under the influence of five different perpetrators of CYP enzymes. Based on the DDI simulations, the following recommendations for OSI dosing adjustments are proposed: (i) when co-administered with FLUV 50 mg OD, no need to adjust OSI dosage (Fig. 5C). (ii) in the presence of ITR 200 mg BID or FLUC 150 mg OD for long time co-administration, a reduction in OSI dosage to 40 mg may be considered, or no need to adjust. (iii) co-administration with RIF or EFA 600 mg OD indicates the need for OSI dose escalation to 160 mg OD.

**Table 4:** The ratio of plasma PK variables change of OSI in DDIs.

Perpetrators	Dosing regimens	Predicted ratios		Observed ratios	
		AUC	C_max_	AUC	C_max_
ITR	OSI: Single-dose of 80 mg OD on days 1 and 10; ITR: Repeated-doses of 200 mg BID from days 6 to 18	1.60	1.06	1.26	0.83
RIF	OSI Repeated-doses of 80 mg OD from days 1 to 29; RIF: Repeated-doses of 600 mg OD from days 6 to 30	0.16	0.40	0.20	0.26
ITR	Concomitantly used at repeated-doses of OSI 80 mg OD with ITR 200 mg BID, FLUC 150 mg OD, FLUV 50 mg OD, RIF 600 mg OD. And EFA 600 mg OD, respectively, for 14 days	2.21	1.60	–	–
FLUC		1.75	1.32	–	–
FLUV		1.41	1.20	–	–
RIF		0.15	0.38	–	–
EFA		0.28	0.50	–	–

–: no data reported.

Furthermore, Fig. 5F,G demonstrate that the plasma C_trough_ of OSI remains within the safe PK threshold at different dosing regimens when concomitantly used with the five perpetrators of CYP metabolizing enzymes. Notably, the classical area AUC ratio method would suggest avoiding co-administration with RIF, or alternatively, dose escalation to approximately threefold higher dosage with EFA. However, the PBPK-EO simulations contradict this classical approach, indicating that dose escalation to 160 mg is appropriate when co-administered with RIF or EFA. These findings are consistent with the proposed clinical dosing strategies^55^.

## Discussion

This study has successfully developed a PBPK-EO model for OSI in patients, enabling the simulation of plasma C_trough_ and the time-course of intracranial EGFR engagement for OSI. The accuracy of the PBPK-EO predictions was validated against nine clinical PK studies (refer to Table 3), and two clinical DDI studies (refer to Table 4). The PBPK-EO model explained the on-target resistance mechanism of OSI to C797S mutation. Moreover, the PBPK-EO model identified three key factors with a significant impact on OSI plasma C_trough_ and intracranial EO_trough_. Additionally, the research determined appropriate dosing regimens for OSI when administered alone and in the context of DDIs with perpetrators of five CYP metabolizing enzymes (Figs. 4, 5). Importantly, the simulation results provide valuable insights into the efficacy of OSI in targeting specific mutations (T790M and L858R) in brain metastases, as well as its limitations regarding resistance mutation (C797S). To the best of our knowledge, this study represents the first attempt to simultaneously simulate the PK and time-course of intracranial EGFR engagement for OSI.

The sensitivity analysis has underscored the significance of albumin levels as sensitive parameters for C_trough_ in plasma and BRT among all selected parameters. Notably, multiple clinical studies have established a strong association between plasma albumin levels and clinical efficacy^56^. Furthermore, the wide variability in albumin levels among patients (ranging from 2.0 to 58 g/L) has been observed^48,49^, potentially leading to substantial variations in plasma exposure and efficacy. Although ABCB1 CL_int,u_ and EGFR T_0_ were not identified as sensitive parameters in the sensitivity analysis, they have been strongly linked to clinical exposure and efficacy^14,53^. Additionally, EGFR overexpression occurred in patients has been reported^57^. Therefore, this study specifically assessed the impact of these three key parameters on plasma C_trough_ and the time-course of intracranial EGFR engagement for OSI. The simulations revealed that these three key parameters exert a significant impact on plasma C_trough_ and intracranial EO_trough_. When these parameters exceed certain values, OSI plasma C_trough_ or intracranial EO_trough_ could compromise PD efficacy or surpass the PK safe threshold (refer to Fig. 3). The observed effects of these key parameters identified by the PBPK-EO model on plasma C_trough_ and intracranial EO_trough_ are consistent with clinical observations^14,53,56^. Overall, the findings from the PBPK-EO model emphasize the substantial influence of these factors on OSI exposure and target engagement in BRT, highlighting their critical role in guiding personalized treatment approaches for patients with brain metastases.

In a recent study, Reddy et al. addressed dosing considerations of OSI when administered concurrently with CYP3A4 inhibitors or inducers^22^. However, their approach primarily relied on the conventional method of determining OSI dosage through plasma OSI AUC ratios. In contrast, our research diverges from theirs in three significant ways: (i) Our study initially developed a PBPK-EO model for OSI in the cancer population, building upon the foundation laid by Reddy et al.’s work. Notably, we refined several parameters such as GET, hematocrit, and albumin levels within our PBPK model to better simulate PK of OSI in patients. (ii) Unlike Reddy et al.’s methodology, which primarily focused on plasma AUC ratios, our investigation targeted patients with brain metastases experiencing DDIs. We determined the optimal OSI dosing regimen by assessing both efficacy, through EGFR occupancy (T790M and L858R mutants), and safety, via C_trough_ in BRT. (iii) Notably, our PBPK-EO model analyzed additional factors influencing PK and EO of OSI, such as ABCB1 CL_int,u,_ albumin levels, and EGFR expression. These variables play crucial roles in determining both intracranial C_trough_ and engagement for OSI, which can provide guidance for personalized medicine.

The simulations indicate that dosing regimens of 80 mg and 160 mg OD, as well as 40 mg and 80 mg BID, are viable options for treating patients with brain metastases. These dosage regimens are shown to ensure that OSI achieves the desired efficacy and safety within the established PK/PD threshold values. Furthermore, the PBPK-EO model suggests that OSI at 160 mg OD can effectively engage T790M/L858R at higher levels and for an extended duration, surpassing the 80% occupancy threshold (refer to Fig. 5), As demonstrated from clinical studies^11^. The PBPK-EO model also provides recommendations for adjusting OSI dosing when co-administered with different perpetrators of CYP enzymes. Specifically, the model suggests an appropriate dosing regimen of 80 mg OD with FLUV, ITR or FLUC for co-administration, and an increase to 160 mg OD with RIF or EFA. The recommendation for dosing adjustment with ITR differs from the clinical trial suggestion^54^. This variance stems from the finding that the AUC ratio of OSI was 1.24-fold higher when co-administered with itraconazole compared to OSI alone, as reported by Vishwanathan et al.^54^. Consequently, adjusting OSI dosing was not recommended due to the AUC ratio being less than 2.0. However, it should be note that in Vishwanathan et al.’s study^54^, ITR was dosed from day 6 to day 18 while OSI was administered only on day 10, resulting in 1 day of co-administration in NSCLC patients. Additionally, the long-term effects of combined administration on OSI exposure were not assessed in their study. Simulations conducted indicate that intracranial T790M/L858R and L858R mutations occupancy can exceed 80% at an OSI dose of 40 mg when co-administration time exceeds 7 days. This observation could potentially explain the disparity between our study and Vishwanathan et al.'s study.

These simulations offer valuable guidance for optimizing OSI dosing regimens in the context of various CYP enzyme perpetrators, supporting informed decision-making for personalized dosing strategies in the clinical management of OSI. Overall, the insights derived from the PBPK-EO model support the selection of an optimal dosing regimen for OSI in the treatment of patients with brain metastases, taking into account both clinical efficacy and safety parameters. These findings provide valuable guidance for designing dosing strategies in clinical practice, striking a balance between therapeutic benefit and risk mitigation.

The current model has several limitations. The primary challenge is the lack of experimentally determined time-profiles of intracranial EO in humans. Hence, the time-course of intracranial EO by OSI has only been validated using observed data from cells (see Fig. 2), which poses a limitation in directly applying the model to human intracranial EO profiles. Secondly, the predicted free concentration of OSI in BRT was only verified using observed concentrations in CSF, which presents a limitation in directly confirming the free concentration of OSI in BRT itself.

## Conclusion

In conclusion, this study has successfully developed and validated a PBPK-EO model for OSI in patient populations. The models are capable of simulating the pharmacokinetic concentration–time profiles and the time-course of EGFR engagement for OSI. Additionally, the study investigated three key factors that significantly influence the PK and PD of OSI. The PBPK-EO model offers valuable guidance for optimizing OSI dosing regimens, whether used alone or in the context of different CYP enzyme perpetrators. These findings provide important insights for personalized dosing strategies and clinical management of OSI, contributing to improved treatment efficacy and safety for patients, particularly those with brain metastases.

### Supplementary Information

Supplementary Information.

Supplementary Figure S1.

Supplementary Tables.

## Data Availability

The study contains original contributions that are detailed in the article and supplementary material. For further inquiries, please contact the corresponding authors.

## Code availability

The mathematical algorithm is detailed through the use of differential Eqs. (3)–(5), which are accessible within the article. These equations can be executed by consulting the supplementary PK-Sim simulation documentation.
