# Supplementary material for: Integrated PBPK-EO modeling of osimertinib to predict plasma concentrations and intracranial EGFR engagement in patients with brain metastases
Source: Sci Rep. 2024 Jun 3;14:12736. doi: 10.1038/s41598-024-63743-z (PMC11148161; doi:10.1038/s41598-024-63743-z)
Supplement: Supplementary file 2 — Supplementary Figure S1. [file 41598_2024_63743_MOESM2_ESM.docx]

**Figure S1. The schematic representation of the PBPK-EO model for OSI.**

The PBPK-EO model encompasses the gastrointestinal tract, the circulatory system (including both arterial supply and venous return), the eliminating tissues (such as the liver and kidneys), as well as other non-eliminating tissues (such as lung and bone). These compartments are connected through blood flow(Q). OSI, the therapeutic agent, undergoes metabolism in the gastrointestinal tract and liver, involving several cytochrome P450 enzymes like CYP1A2, CYP2A6, CYP2C9, CYP2E1, CYP3A4, and CYP3A5. Within the model, the brain is a specialized compartment that includes not only the brain tissue but also sub-compartments referred to as plasma, blood cells, interstitial and intracellular spaces. The BBB, a crucial structure within this region, is equipped with specific membrane transporters known as ABCB1 and BCRP, which regulate the passage of substances into and out of the brain. The PBPK-EO model is also used to simulate the concentration of free OSI within the BRT. Additionally, the model simulates the occupancy of the EGFR by OSI within the intracellular compartment.
