# Supplementary material for: Integrated PBPK-EO modeling of osimertinib to predict plasma concentrations and intracranial EGFR engagement in patients with brain metastases
Source: Sci Rep. 2024 Jun 3;14:12736. doi: 10.1038/s41598-024-63743-z (PMC11148161; doi:10.1038/s41598-024-63743-z)
Supplement: Supplementary file 3 — Supplementary Tables. [file 41598_2024_63743_MOESM3_ESM.docx]

**Supplementary Table S1** The ratio of OSI (80 mg OD) PK variables with or without ABCB1 and BCRP transport

| Variables | OSI with ABCB1 and BCRP | OSI without ABCB1and BCRP | Predicted ratio ^a^ | Observed ratio ^b^ |
| --- | --- | --- | --- | --- |
| C_max_ (nmol/L) | 175.4 | 233.8 | 1.33 | 1.54 |
| AUC(nmol·h/L) | 8975 | 12943 | 1.44 | 1.40 |

^a^: Calculated using ratios of the data without ABCB1 or BCRP to those with ABCB1 or BCRP. ^b^: Available from the data in mouse.

**Supplementary Table S2** The inhibition and induction parameters of five perpetrators

| Perpetrators | Interaction mechanism of co-medication | CYP3A4 K_i_ (μM) | CYP1A2 K_i_ (μM) | CYP2C9 K_i_ (μM) | CYP2C9 | | CYP3A4 | |
| --- | --- | --- | --- | --- | --- | --- | --- | --- |
|  |  |  |  |  | EC_max_ | EC_50_(μM) | EC_max_ | EC_50_(μM) |
| Itraconazole (inhibitor, ITR) | Strong CYP3A4 inhibitor | 0.0013 | - | - | - | - | - | - |
| Hydroxy-itraconazole |  | 0.0023 | - | - | - | - | - | - |
| Fluconazole (inhibitor, FLUC) | Moderate CYP3A4 and CYP2C9 inhibitor | 16.6 | - | 6.5 | - | - | - | - |
| Fluvoxamine (inhibitor, FLUV) | Strong CYP1A2 and Moderate CYP3A4 inhibitor | 0.052 | 0.011 | - | - | - | - | - |
| Rifampicin (inducer, RIF) | Strong CYP3A4 and moderate CYP2C9 inducer | - | - | - | 2.41 | 0.64 | 9.0 | 0.34 |
| Efavirenz (inducer, EFA) | Moderate CYP3A4 inducer | - | - | - | - | - | 5.2 | 0.07 |

**Supplementary Table S3** Sensitivity analysis results

| Modelling parameters | SC values | | | |
| --- | --- | --- | --- | --- |
|  | Plasma C_trough_ | Intracranial C_trough_ | EO_trough_ for T790M | EO_trough_ for L858R |
| f_up_ | -1.29 | -1.18 | 0.03 | 0.03 |
| Albumin | -1.83 | -1.71 | 0.04 | 0.04 |
| CYP1A2 CL_int,u_ | -0.16 | -0.26 | - | - |
| CYP2C9 CL_int,u_ | -0.25 | -0.36 | - | - |
| CYP3A4 CL_int,u_ | -0.52 | -0.43 | - | - |
| ABCB1 CL_int,u_ | -0.58 | -0.35 | - | - |
| BCRP CL_int,u_ | -0.012 | -0.01 | - | - |
| k_on_ T790M | - | - | -0.1 | - |
| k_on_ L858R | - | - | - | 0.07 |
| k_off_ | - | - | -0.02 | -0.02 |
| EGFR T_0_ | - | - | -0.08 | -0.1 |
| k_deg_ EGFR | - | - | -0.02 | -0.02 |
